# What makes palliative care needs “complex”? A multisite sequential explanatory mixed methods study of patients referred for specialist palliative care

**DOI:** 10.1186/s12904-020-00700-3

**Published:** 2021-01-15

**Authors:** Anne M. Finucane, Connie Swenson, John I. MacArtney, Rachel Perry, Hazel Lamberton, Lucy Hetherington, Lisa Graham-Wisener, Scott A. Murray, Emma Carduff

**Affiliations:** 1 Marie Curie Hospice Edinburgh, 45 Frogston Road West, Edinburgh, EH10 7DR UK; 2 Primary Palliative Care Research Group, Usher Institute, University of Edinburgh, Teviot Place, Edinburgh, EH8 9AG UK; 3 Unit of Academic Primary Care, University of Warwick, Gibbert Hill, Coventry, CV4 7AL UK; 4 Marie Curie Hospice West Midlands, Marsh Lane, Solihull, B91 2PQ UK; 5 Marie Curie Hospice Belfast, 1A Kensington Road, Belfast, BT5 6NF UK; 6 Marie Curie Hospice Glasgow, 133 Balornock Rd, Glasgow, G21 3US UK; 7 Centre for Improving Health-Related Quality of Life, School of Psychology, Queen’s University Belfast, Belfast, BT7 1NN UK

## Abstract

**Background:**

Specialist palliative care (SPC) providers tend to use the term ‘complex’ to refer to the needs of patients who require SPC. However, little is known about complex needs on first referral to a SPC service. We examined which needs are present and sought the perspectives of healthcare professionals on the complexity of need on referral to a hospice service.

**Methods:**

Multi-site sequential explanatory mixed method study consisting of a case-note review and focus groups with healthcare professionals in four UK hospices.

**Results:**

Documentation relating to 239 new patient referrals to hospice was reviewed; and focus groups involving 22 healthcare professionals conducted. Most patients had two or more needs documented on referral (96%); and needs were recorded across two or more domains for 62%. Physical needs were recorded for 91% of patients; psychological needs were recorded for 59%. Spiritual needs were rarely documented. Referral forms were considered limited for capturing complex needs. Referrals were perceived to be influenced by the experience and confidence of the referrer and the local resource available to meet palliative care needs directly.

**Conclusions:**

Complexity was hard to detail or to objectively define on referral documentation alone. It appeared to be a term used to describe patients whom primary or secondary care providers felt needed SPC knowledge or support to meet their needs. Hospices need to provide greater clarity regarding who should be referred, when and for what purpose. Education and training in palliative care for primary care nurses and doctors and hospital clinicians could reduce the need for referral and help ensure that hospices are available to those most in need of SPC input.

**Supplementary Information:**

The online version contains supplementary material available at 10.1186/s12904-020-00700-3.

## Background

People with advanced illness should be referred to specialist palliative care (SPC) services if they have needs that cannot be addressed by usual care. Criteria for specialist palliative care referral include a diagnosis of advanced cancer, physical symptoms, low performance status, psychosocial distress, advance care planning needs, family concerns and patient request [1]. However a consensus on referral criteria is lacking, and access to specialist palliative care is determined by the existence of any of these criteria, rather than the level of complexity of need [2–4].

In the United Kingdom, palliative care is provided through both specialist and generalist services. Specialist palliative care services are those offered by multidisciplinary National Health Service (NHS) teams or hospices employing staff with the requisite qualifications and expertise to support terminally ill people and their families. Most inpatient and community specialist palliative care is provided by hospices [5, 6], which are charity-based localised services funded mainly through charitable donations [7]. Hospices offer a wide range of services, free-of-charge, to address the physical, psychological, social and spiritual needs of people with a terminal illness and their families. These can be inpatient, community-based or can involve attending the hospice as an outpatient or day patient. Hospices are evolving and have shifted their focus from caring for patients with cancer to the development of services for all terminally ill patients; while also seeking to offer services earlier in the illness trajectory when needed. As hospice services have developed to suit the needs of their local population and receive only partial statutory funding through local commissioning processes, there is much variability in the services offered [7–9].

The term ‘complex need’ is frequently used to describe the needs of patients accessing specialist palliative care, including hospice care. There is no standard definition of complexity in palliative care, nor a distinct set of needs that are understood as ‘complex’ [4, 10]. Rather, qualitative studies have identified potential indictors of complex needs, including number, severity and changing nature of need, alongside the interaction of multiple needs across different domains (physical, psychological, social and spiritual) [4, 10, 11]. Communication challenges, learning disabilities and multimorbidity may increase the complexity of need [11].

At a broader systems level, dissonance in relationships between the patient, their family and/or healthcare professionals can impact complexity [4, 11]. Lack of engagement with services, sometimes as a result of potentially stigmatising pre-existing mental health issues or diseases, increase ‘invisible’ complexity [4]. Lack of confidence amongst some primary care professionals in caring for patients approaching end-of-life can lead to judgements that care needs are complex, whereas professionals with more experience might not consider such needs complex, highlighting the subjective nature in making judgements about complexity of need [11].

Researching complexity-informed approaches needs to account for the dynamic contexts, unpredictable process, and self-organizing objects (such as continuous adaptations initiated by frontline staff to allow them to complete tasks, given local demands), that disrupt the linear pathways of traditional medical care and research [12, 13]. As a practice-based starting point to inform our understanding of complex needs in the ecology of hospice referral processes, and to inform the development of guidance, we sought to describe the documented needs of patients referred by primary and secondary care professionals to a hospice service. As referrals of complex needs are emergent and dynamic “events in systems”, [14] we then looked to explore staff perspectives on this process.

## Methods

### Design

We conducted a mixed methods study consisting of a retrospective case note review and qualitative data collection via focus groups. We adopted an explanatory sequential mixed methods design. This type of mixed methods design occurs in two distinct phases, starting with the collection and analysis of quantitative data, followed by the collection and analyses of qualitative data to expand on quantitative results collected in the first phase [15].

### Setting

Data were collected in four hospices across three UK nations – Scotland (2 sites), Northern Ireland (1 site), and England (1 site). All offered hospice inpatient services and day therapies; three offered community palliative care Clinical Nurse Specialist (CNS) services (Hospices 1, 2 and 4); and two offered outpatient clinics (Hospices 2 and 4). There was variability in how services were organized within each hospice. For instance, in one hospice, day therapies were part of the overall community nursing service, whereas in others day therapies was a separate service. The interventions offered within day therapies also varied as has been described elsewhere [8].

### Retrospective case note review

#### Participants

An automated list of all consecutive new referrals between June and December 2017 was generated at each hospice. All referrals were eligible for inclusion unless the referral forms and related correspondence was missing or incomplete. A sample of approximately 240 was deemed feasible and appropriate to allow descriptive analysis across sites, in line with previous studies [16].

#### Data collection

We reviewed referral documentation, including referral forms and documented phone or written correspondence with the referrer, which occurred prior to contact with the patient. The format of referral forms varied across settings. Data from referral documentation were abstracted using a standardised form, developed specifically for this study, which listed indicators of complex need identified from the research literature (Supplementary material 1). Four clinicians (CS, RP, LH, HL) with experience of local referral processes undertook data collection and abstraction at their respective site (target of approximately 60 records at each site). Training in data abstraction was provided by CS and regular discussion with the wider team ensured consistency across sites. Data were abstracted directly from the referral documentation to the standardised form and recorded in Microsoft Excel. To ensure the quality of data, we implemented the strategies proposed by Gilbert et al. [17] for case note review: training of data abstractors, explicit case selection, precise definition of variables, use of standardised abstraction forms, routine meetings to review progress, and monitoring performance of data abstractors. It was not possible to blind abstractors to the aim of the study; nor for inter-rater agreement to be tested on all data collected due to resource constraints which allowed for only one abstractor per site. However, this was done on a subset where ambiguity existed.

#### Variables

Key variables included whether the referral form documented physical needs (e.g. pain, shortness of breath, confusion, fatigue); psychological needs, spiritual needs, functional care needs, social care needs, planning and end of life care or communication needs (Yes/No). We also extracted data on patient characteristics, primary diagnosis, source of referral and service first referred to.

#### Bias

To minimize the risk of selection bias, random numbers were assigned to each referral and the first 60 referrals, in numeric order, were analysed at each site. Referrals containing too few data for analyses were excluded. Measurement bias was managed by selecting data abstractors at three sites who were separate to those involved in data analysis.

#### Data analysis

Data were analyzed descriptively using EXCEL and SPSS version 24. Variables were compared across all sites.

### Focus groups

#### Participants

We conducted four focus groups – one at each site. A purposive sample of staff from each hospice was invited to participate, to include representatives from the medical, nursing, allied health professional and administration teams. All received a participant information sheet and signed a consent form in advance of participation.

#### Data collection

A member of the research team (CS; LGW; JM; LH) with qualitative research training facilitated the focus group at each site. Two facilitators were hospice doctors working at the focus group sites (CS and LH). Two were academic researchers known to participants (LGW and JM). During each focus group, the facilitator presented key findings from the case note review (e.g. source of referrals, number and type of needs documented on referral forms) and facilitated the discussion using a semi-structured interview schedule (Supplementary material 2). Focus groups lasted 1 to 1.5 h, were audio-recorded and transcribed.

#### Data analysis

Transcriptions were analysed using a constant comparison approach by one member of the research team (JM) [18], reviewed by three others (AF, CS, RP) and then verified by the wider team. The research team agreed the data contained sufficient “information power” - which takes into account (a) the aim of the study, (b) sample specificity, (c) the evolving nature of complexity science and theory to which the study will contribute, (d) the descriptively rich quality of dialogue, and (e) analysis strategy – for the purposes of this study [19].

#### Public and patient involvement (PPI)

A member of the Marie Curie Voices group, a group of patient and carer representatives with experience of palliative care, provided feedback on the findings, which in turn informed the discussion.

#### Ethical and governance considerations

The South East Scotland Research Ethics Committee confirmed that this study was a service evaluation as opposed to research study, and thus external ethics approval was not required. We obtained approval from the Research Governance Committee at each hospice site. The study is reported according the Good Reporting of a Mixed Methods Study (GRAMMS) reporting guidance for mixed method studies (Supplementary material 3) [20].

## Results

### Retrospective case note review

Documentation for 239 referrals across four hospice sites was examined (49% female; 51% male). Mean age of patients was 72 years (range: 22–97 years) and the majority had a primary diagnosis of cancer (87%).

Source of referral varied by hospice (Table 1). Across all hospices, most referrals came from hospital, with a third coming from general hospital teams (*n* = 78, 33%), and just under a third from hospital SPC teams (*n* = 70, 29%). Just under a third were from GPs (*n* = 71, 30%). New referrals were most frequently received by the community clinical nurse specialist (CNS) hospice team where such a service existed (56% of all referrals). 23% of all new referrals were for the inpatient unit and 19% for day services. Hospice 3 did not run a community specialist palliative care CNS service, so most referrals were for day therapies. At Hospice 1, day therapies are provided by the community team, so most referrals were initially directed there. Across all hospices, 89% of all referrals were accepted. Largely, a referral was not accepted because the patient declined the service or died prior to assessment.

**Table 1:** Overview of patient referrals by hospice site

Hospice	Number of referrals analysed	Source of referral^a^	Hospice service referred to	Cancer as primary diagnosis %	Male %	Female %	Age Mean (range)
1	60	48% Hospital 42% GP 5% Hospital SPC 5% Hospital and GP	83% Community 15% Inpatient 2% Day therapies	88%	48%	52%	72 (48–93 yrs)
2	62	39% Hospital 29% GP 31% Hospital SPC 2% Hospital SPC and GP	89% Community 10% Inpatient 2% Day therapies	90%	55%	45%	74 (37–93 yrs)
3	60	5% Hospital 20% GP 57% Hospital SPC 18% Community SPC	0% Community 48% Inpatient 52% Day therapies	95%	48%	52%	69 (30–94 yrs)
4	57	39% Hospital 28% GP 25% Hospital SPC 2% Hospital SPC & GP 2% Community SPC 5% Hospital and GP	51% Community 21% Inpatient 21% Day therapies 7% Unknown	74%	54%	46%	74 (22–97 yrs)
**All sites combined**	**239**	**33% Hospital** **30% GP** **29% Hospital SPC** **1% Hospital SPC & GP** **5% Community SPC** **3% Hospital and GP**	**56% Community** **23% Inpatient** **19% Day therapies** **2% Unknown**	**87%**	**51%**	**49%**	**72** **(22–97 yrs)**

^a^Notes: ‘Hospital’ excludes the hospital specialist palliative care team. Hospital SPC means referral from the hospital specialist palliative care team. Percentages may not add to 100% due to rounding

#### Patient needs documented at the time of referral

**Fig. 1:**
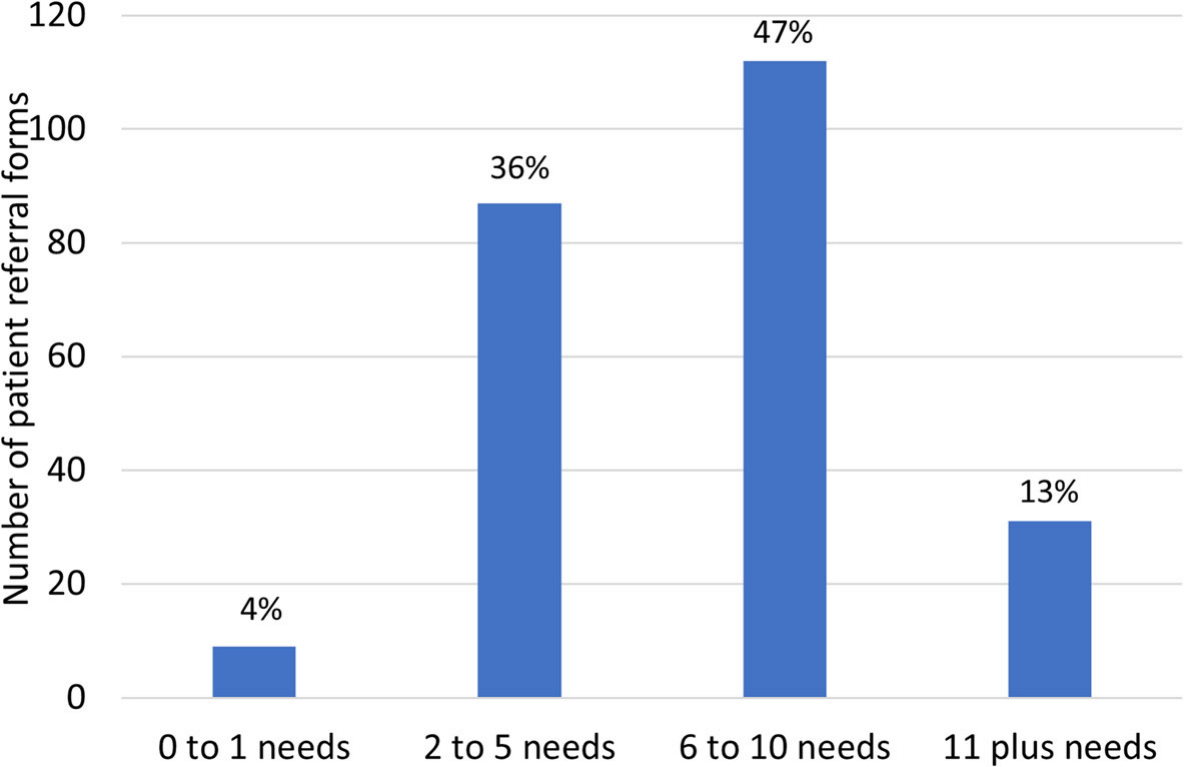
Number of specific needs documented on referral forms (*n* = 239)

**Fig. 2:**
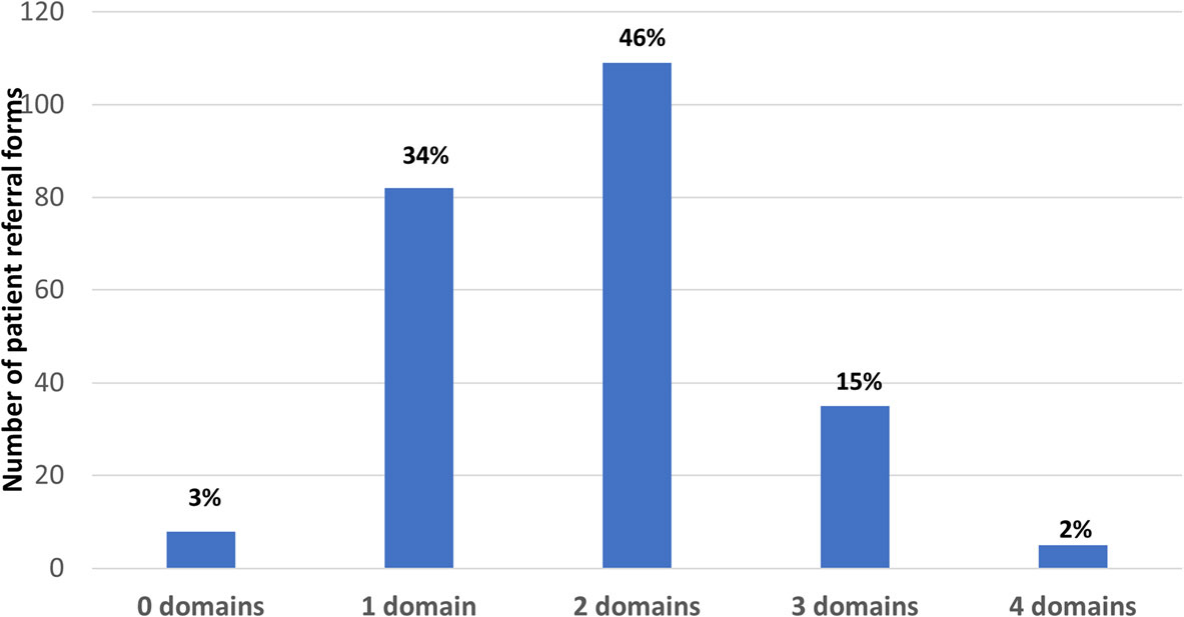
Number of broad domains of need (physical, psychological, social or spiritual) documented by referrer (*n* = 239)

Overall, 230 patients (96%) had two or more needs documented on referral (Fig. 1). This included 59% who had six or more distinct needs documented. For 149 (62%) of patients, needs were documented across two or more broad domains of need – physical, social, psychological, or spiritual (Fig. 2). Eight patients were referred with needs considered separate from the four domains (e.g. end of life care or functional care needs).

**Fig. 3:**
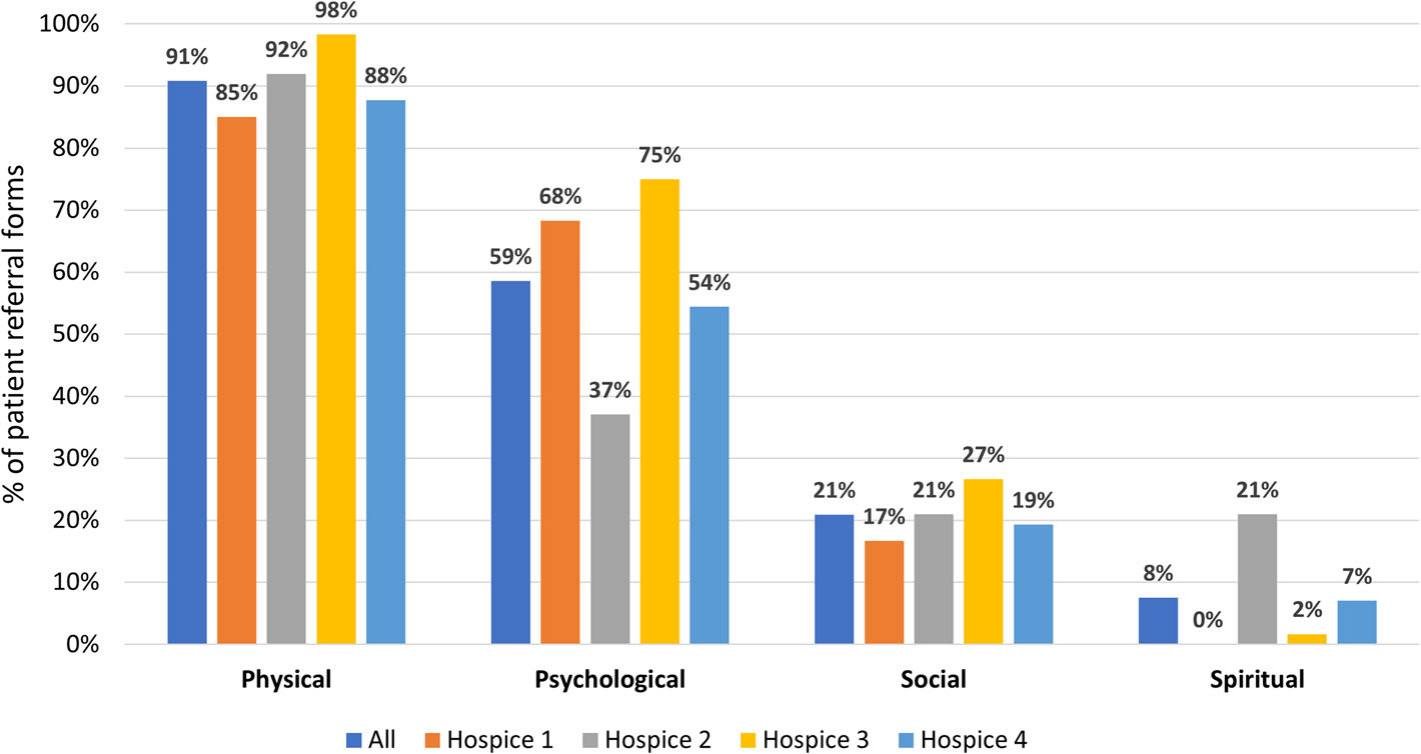
Broad domains of need documented in referral documentation (*n* = 239)

**Fig. 4:**
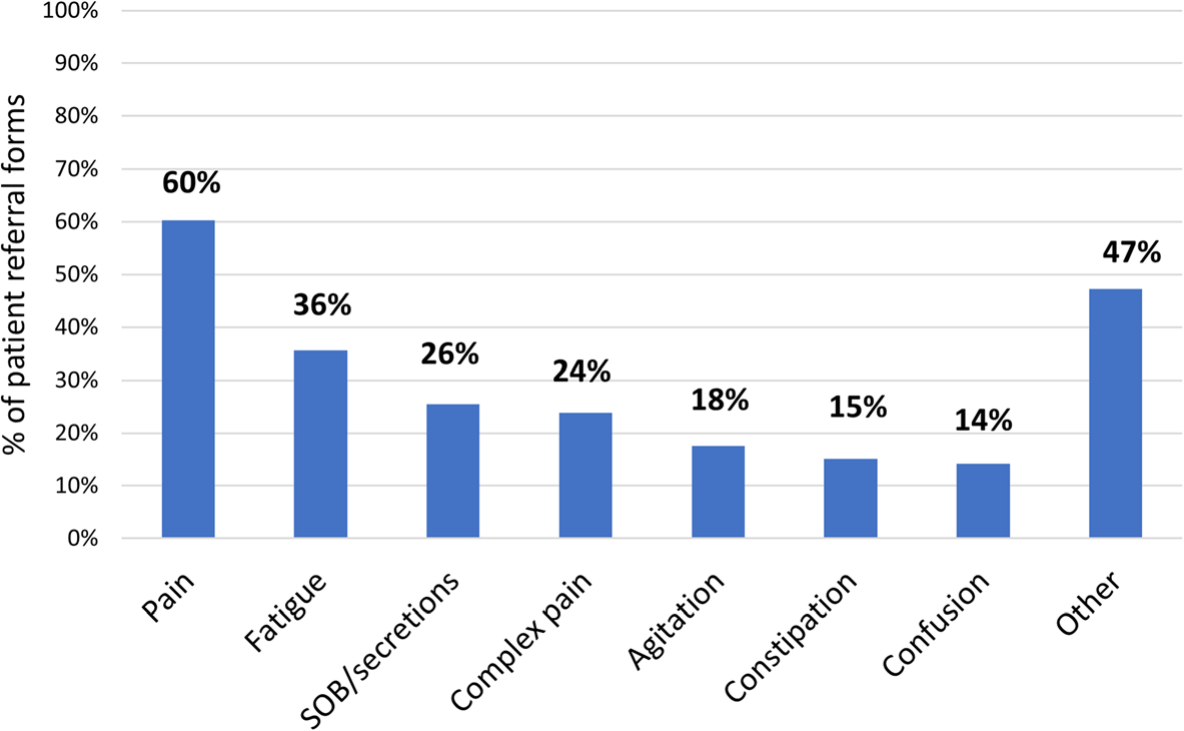
Breakdown of physical needs documented by referrer (*n* = 217)

**Fig. 5:**
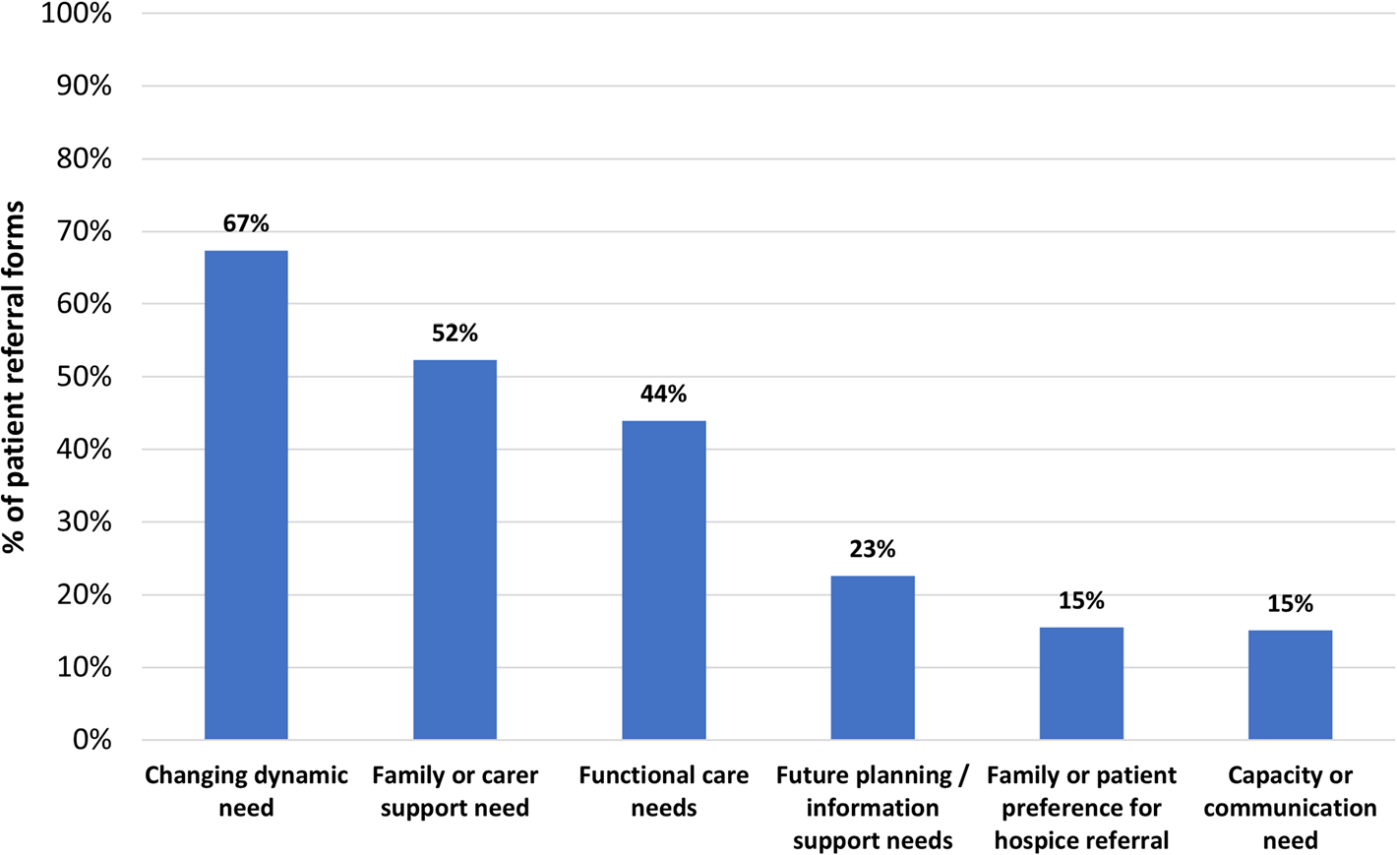
Breakdown of other needs documented by referrer (*n* = 239)

Physical needs were nearly always documented (Fig. 3). Pain was most frequent (*n* = 144, 60%) followed by fatigue (*n* = 85, 36%) (Fig. 4). Complex pain was specifically mentioned for 57 patients (24%). Psychological needs were noted for 140 patients (59%) but were not generally specified further. Social needs were documented on 50 referral forms (*n* = 21%), and included needs associated with caring responsibilities (*n* = 20), social isolation (*n* = 15) and housing concerns (n = 8). Spiritual needs were noted in only 8% of referral forms. Other needs documented included: rapidly changing needs (67%); family or carer support needs (52%) and functional care needs (44%) (Fig. 5).

#### Variation in documentation of needs by site

Across all locations, the same overall patterns existed, with physical needs most often documented, followed by psychological, social and spiritual needs in that order. However, variation was also evident (Fig. 3). Most notably, psychological needs were documented on referral for 37% of patients referred to Hospice 2, but 75% of patients referred to Hospice 3. Overall, spiritual needs were documented for 8% of all newly referred patients, but this varied from 0% in Hospice 1 to 21% in Hospice 2.

### Qualitative findings

Twenty-two participants took part in focus groups across the four sites (Table 2).

**Table 2:** Focus Group Participants

Site	Total participants	Doctors	Clinical Nurse Specialist	Nurses (inpatient units)	Allied health professionals	Administrators	Medical Students
**1**	4	1	2	1	0	0	0
**2**	7	2	1	2	1	0	1
**3**	4	1	0	2	1	0	0
**4**	7	4	1	0	1	1	0
**Total**	**22**	**8**	**4**	**5**	**3**	**1**	**1**

Six themes were identified across the focus groups: i) Prioritisation of physical needs; ii) Referral forms as limited tool; iii) Referrals associated with resource constraints, iv) Interpreting a referral form; v) Tension in accepting early versus later referrals vi) Referrals of people with cancer predominate.

#### Prioritization of physical needs

Participants reported that physical needs were generally prioritized on referral documentation, because these symptoms were most readily recognized, quantifiable or perceived as most likely to result in referral. Psychological, social, and other care needs were less likely to be documented.

*‘some doctors.. just focus on pain … and don’t see the rest of the symptoms’ (FG Site 1).*

*‘often the GP, like you say, will put something down, pain, but that might not be their major problem it could be something you know social, family’ (FG Site 4).*

*“Physical symptoms are much.. more quantifiable than maybe psychological distress or psychological symptoms or care needs.. ...so that’s it’s easier to get across in a referral.” (FG Site 2).*

#### Referral forms as limited tools

Referral forms were perceived as limited in capturing patient needs and associated complexity. While referrals were generally perceived as appropriate – in the sense that those referred benefited from the referral - often the needs documented on the referral form did not align with those identified on first assessment.

*“I think with a lot of our patients you can’t capture them on paper” (FG Site 4)*

Referral forms were considered the first step to further assessment where patient’s needs could be captured fully.

*“I think because of the complexity of it, the only thing we could have done was go out and actually go on the ground and see what [the situation] was” (FG Site 2)*

*“ … when you go out it’s [the referral] absolutely appropriate, but not appropriate because of the reason that the GP or any referrer thought it was appropriate for. It’s because you’ve gone out, you’ve spent that time and you’ve uncovered a lot more than actually what was in the referral” (FG Site 1)*

Comparisons were made between different referral forms used, including the use of free text and tick boxes, but no consensus was reached as to which was preferred. Participants agreed that forms should capture essential information (although not what this should be), be simple to complete and not be expected to capture everything about a patient.

*“The thing I don’t like about ours [referral form]..there’s so many little boxes to tick and there’s not enough room just for free-text. Ticking the boxes doesn’t always give you what you need to know." (FG Site 3)*

#### Referrals associated with resource constraints

Participants acknowledged the subjective nature of the judgement that a patient needs to be referred for hospice care. Sometimes this is due to a lack of resources or a referrers lack of confidence in addressing palliative care issues in their own setting:

*“whatever form you use, the complexity that goes on the form will be the perception of complexity from the person writing the form … so if you’ve got somebody who doesn’t like [palliative care], finds it really uncomfortable and doesn’t want to talk about DNACPR that will probably come through on the form that actually they’re [the patient] really tricky and they don’t want to discuss advance care planning”. (FG Site 4)*

Lack of resources and time pressures locally may also result in a referral to specialist palliative care:

*“...the pressures that they’re [GPs] getting, I think they’re under increasing demand and I think they see the specialist palliative care service as a resource.” (FG Site 1)*

#### Interpreting a referral form

Referral forms were perceived by hospice clinicians as a limited tool, of variable quality, beset by multiple tensions inherent in providing services for patients with complex needs. Thus interpreting the form became an important skill. For example, some participants reflected that the information referrers provided was influenced both by a referrer's lack of knowledge about specific hospice services *and* by a desire for the referral to be accepted.

*“the referrer is trying to essentially sell you a patient so that you take them on and if they don’t, you know, if they don’t use the right buzzwords or use the right kind of things and you know, they know that we’re going to say no” (FG Site 2)*

Referrers were sometimes thought to emphasise certain traits (e.g. physical symptoms) they thought would result in successful referral, as well as downplay other issues (e.g. social or family problems). Staff involved in triaging needed to decipher what service was most appropriate.

*“. . . that [is the] complexity of the triaging process and the skill of the triage person . . . you’re triaging calls because you’re getting referrals from everybody wanting beds on an inpatient unit and you’re trying to prise out ‘well what is it for and is it appropriate?’” (FG Site 1)*

#### Tension in accepting early versus later referrals

Tensions were experienced when considering early referrals of patients with potentially complex needs. Participants described difficulties managing finite resources, balancing early intervention with focusing on complex needs, and the evolving expectations of hospices (e.g. to care for more people with non-malignant disease and offer specialist palliative care earlier). One tension was a recognition that hospice services could benefit most patients but was a finite resource that had to be allocated effectively.

*“We still haven’t worked it out [balancing resource and demand], I still don’t think palliative care have worked out how we’re going to manage” (FG Site 4)*

Similarly, participants described the tension between prioritising more patients with complex needs and being involved with patients earlier to prevent or lessen future complexity.

*“[We] advised that we come, so that we get to know you for later on down the line, which isn’t a bad idea either” (FG Site 3)*

#### Referrals of people with cancer predominate

Hospice referral is still generally perceived as appropriate for anyone with advanced cancer, irrespective of their symptoms control needs:

*“I don’t think it’s based on need, I think it’s probably a perception, still a perception, that palliative care is for people with cancer because often people are referred with cancer before they have any symptoms at all, but they’ve been given a diagnosis of terminal cancer.” (FG Site 2).*

People with a non-cancer diagnosis were perceived to be less likely to be referred, possibly as their physical symptoms tend to be managed by other services. If they are referred for specialist palliative care, this is often for psychosocial support over a longer period of time:

*“I think that often the non-cancer referrals are more to do with psychological stress and carer stress and anxiety as opposed to physical symptoms” (FG Site 2).*

Participants acknowledged that hospice models of care for those with advanced disease other than cancer were still developing, and presented a challenge:

*“ … non-malignant patients, they are normally longer-term patients so they need less intense [involvement] maybe over a period of time, so we’ve got to change our model and we’re still struggling with that … ” (FG Site 4).*

## Discussion

Previously described markers of complex need were evident in the referral documents of nearly all new patients referred to four hospice services. The vast majority had two or more needs documented; and for most, needs were recorded across two or more domains (physical, social, psychological, or spiritual). Changing dynamic needs were noted for over two-thirds of patients, and family or carer support needs recorded for half. However, complexity was hard to detail or objectively define based on referral documentation alone. Hospice staff perceived referral documents as limited tools, often prioritizing information on physical symptoms over other concerns. Referrals were viewed as influenced by the experience and confidence of the referrer and the resources available to them to directly meet the patients’ needs and diagnosis. Referrals of those with non-malignant disease were far less frequent compared to referrals of those with cancer, and hospice models of specialist palliative care for support for this group still present challenges.

It was evident that for hospice staff, the care of patients with complex needs was intrinsic to their job but was not something easily described or understood. Although referral documentation indicated complex needs for most patients, staff perceived standardised referral forms as limited, containing information of variable quality that needed skilled interpretation to ensure patients' needs could be met. The reliability of the referrer and completeness of referral information has previously been described as a source of uncertainty or bias; and lack of knowledge or experience may over or underestimate actual palliative care need [21]. Language and lack of clear terminology is also a barrier, for instance ‘dying’ can indicate a person recently diagnosed with a terminal illness, or someone approaching end of life [21, 22]. Our study found that language was sometimes used selectively to make a case for referral, whereby the referrer chose words or documented symptoms to make a stronger case for referral, and omitted others as less influential. Participants recognised the initial referral as only the start of a process, requiring further communication between the referrer and provider, culminating in the first assessment.

Physical needs were noted in 91% of referrals, and psychological needs in 59%. Physical needs were generally specified, with pain and fatigue most often documented. These symptoms are typical amongst those approaching end of life [23]; though other common symptoms such as constipation [24] appeared less frequently. Psychological symptoms were typically unspecified and lacked detail. This was because some referral forms provided structured YES/NO boxes to indicate ‘psychological support’. There is a clear need to go beyond the use of generic terms such as ‘psychological support’ and ‘emotional support’ when describing psychological needs of people with a terminal illness. Specific needs relating to anxiety, depression, anger, avoidance, collusion, and anticipatory grief alongside pre-existing mental health disorders are common and should be identified to enable appropriate support. Adding the results of screening tools for psychological problems (e.g. anxiety and depression), could further improve the quality of the referral. Social needs relating to social isolation, caring responsibilities, housing concerns or ‘other’ were noted for one-fifth of patients newly referred; however when patient and family support needs are added, nearly two-thirds of all newly referred patients had social needs documented. Tools such as the Carer Support Needs Assessment Tool (CSNAT) can be used to identify specific carer support needs [25–27], and could enrich the quality of information on referral. Spiritual needs, in the broadest sense, were rarely documented, despite being important for patients and their families [28–31]. This may be due partly to the inclusion of an explicit section about spiritual support needs on some but not all referral form templates. Including an open section on spiritual support needs on referral forms would allow an indication of the importance of spiritual support for the terminally ill person and would help ensure that the person is directed towards the hospice service(s) most aligned with their needs. Our PPI representative noted that the term ‘spiritual need’ should also be defined on referral forms, so that professionals, patients and families have a shared reference point.

Resource or capacity constraints in primary or secondary care settings were perceived to influence whether a SPC referral was made – with less capacity increasing the likelihood of referral. Where there is a discrepancy between the care needs of the patient and the capacity of their care providers to meet their needs (e.g. due to lack of experience, skills or time), patient needs may increase, leading to a referral to SPC services [4, 21]. Cumulative needs [4], which we show are common amongst people with a terminal illness, can be difficult to address within the short space of time available for a primary or secondary care consultation. Lack of confidence or experience in providing palliative care support, for instance prescribing or advance care planning [32], may increase perceived complexity and referral for SPC [11].

Our study highlighted ambivalences or tensions regarding the timeliness of hospice intervention alongside dilemmas about who was best placed to assess and respond. Palliative care is an approach applicable early in the course of a life-threatening illness or severe illness [33, 34]. However, referral for SPC including hospice care tends to occur in the late stage of advanced illness [5, 35]. Staff recognised that complex needs could occur earlier, or could be prevented with earlier intervention, though the capacity implications of offering services at an earlier stage was a concern. Research shows that quality of life of people with a terminal illness oscillates over time, and for some, distress peaks on diagnosis or recurrence [36]. Models of early hospice support need to be developed and evaluated so that people can access SPC when their needs are greatest, irrespective of their prognosis.

### Implications

Uncertainty around what complex needs are and ambivalence regarding the hospice services available are features of the current system. Despite this, we found that “complex needs,” specifically multiple needs within and across domains, are recorded in hospice referrals, though detail is often lacking. Several steps could be taken to improve the consistency of referrals. Referrers may have a history with patients, and could draw more on this knowledge when documenting the reasons for referral to ensure that the patient and their family is directed to the service that best meets their needs. Greater consideration of the non-physical needs of patients is warranted. Across all domains, where appropriate, the use of standardised screening tools and performance measures (e.g. Karnofsky Performance Status; Phase of Illness; Distress Thermometer) as a supplement to free-text information, could provide greater clarity and enable hospices to individualize services early on. Hospices could improve the referral process by ensuring that referrers are aware of the needs addressed by each available service. Palliative care specialists could offer training and support to GPs, community nurses, care-home nurses and other staff to reach all patients in need, especially those with non-malignant disease. Structured referral forms – now normal practice in all other specialties - could contain a section on palliative care provided prior to referral, clarifying what palliative care has already been offered, when and why the person is now being referred for hospice care.

Hospices are increasingly under pressure to show their ‘worth’ to commissioning groups through tangible outputs and impacts, which may contribute to a greater emphasis on more medical aspects of palliative care, which downplays the psychological, social and spiritual care provided. This may partly explain the emphasis on physical symptoms found in referral documentation. Clear communication on the interventions offered by hospices to address non-physical care needs is needed to ensure that referrers and commissioners understand the range of SPC services available, and how SPC can significantly improve quality of life for those with greatest need.

Further research is needed to develop and evaluate referral documentation that is useful and informative to both referrers and hospice service providers. We only analysed needs of those referred to hospice; future work might usefully compare the needs of those referred and those who were not referred, so that care trajectories are better understood. The ID-Pall tool has recently been developed to distinguish between needs that can be provided by non-SPC providers versus SPC [37], further validation and testing in diverse settings is now required.

### Strengths and limitations

These findings relate specifically to the hospices involved in the study, and the results are not generalizable. However, this study highlights the variation in hospice service structure and the documented needs of patients referred to each hospice. The inclusion of four sites, in three regions of the UK allowed exploration of variation. Focus groups consisted of participants from hospice settings, not primary or secondary care settings, though their inclusion in future related studies is recommended.

## Conclusion

Complexity was hard to detail or to objectively define based on referral documentation alone. Given increased complexity of need [38], longevity in prognosis and evidence that early interventions may ameliorate long terms problems, hospices need to provide greater clarity regarding who should be referred, when and for what reason. In the meantime, hospices can improve the referral process by specifying what hospice services are available to meet which needs; communicating regularly with referrers; and providing education and training to support referrers to meet more palliative care needs directly.

## Supplementary Information

**Additional file 1.**

## Data Availability

Data will be made available upon request from the corresponding author.
