# Supplementary material for: What makes palliative care needs “complex”? A multisite sequential explanatory mixed methods study of patients referred for specialist palliative care
Source: BMC Palliat Care. 2021 Jan 15;20:18. doi: 10.1186/s12904-020-00700-3 (PMC7809819; doi:10.1186/s12904-020-00700-3)
Supplement: Supplementary file 1 — Additional file 1. [file 12904_2020_700_MOESM1_ESM.docx]

**Supplementary material 1: Data extraction template**

**Documented markers of complexity**

| **Marker of Complexity** | | Y/N |
| --- | --- | --- |
| **Domain** | **Individual marker of complexity** |  |
| **1. Physical Symptoms** | 1.1 Physical Symptoms ANY |  |
|  | 1.2 Pain |  |
|  | 1.3. Complex Pain |  |
|  | 1.4 Breathlessness / Secretions |  |
|  | 1.5 Nausea/ vomiting |  |
|  | 1.6 Confusion |  |
|  | 1.7. Agitation |  |
|  | 1.8 Constipation |  |
|  | - 1. Fatigue |  |
|  | 1.10 Other (state what) |  |
| **2.Psychological, Emotional or Spiritual Needs of Patient** | 2.1 Psychological / Spiritual/ Emotional Need ANY |  |
|  | 2.2 Psychological/ Emotional Need of Patient |  |
|  | 2.2 Spiritual Need of Patient |  |
| **3. Active Co-morbidities** | - |  |
| **4. Functional Care Needs** | - |  |
| **5. Social Situation** | 5.1 Social ANY |  |
|  | 5.2 Social exclusion |  |
|  | 5.3 Social responsibilities |  |
|  | 5.4 Housing |  |
|  | 5.5 Other social factor (state what) |  |
| **6. Capacity or Communication Need of Patient** | 6.1 Capacity / Communication ANY |  |
|  | 6.2 Capacity |  |
|  | 6.3 Communication |  |
| **7. Patient Characteristics** | 7.1 Patient Characteristics ANY |  |
|  | 7.2 Patients coping mechanisms |  |
|  | 7.3 Patient behaviours |  |
| **8. Patients’ Personal Relationships** | 8.1 Patients’ Personal Relationships ANY |  |
|  | 8.2 Dissonance in relationships |  |
|  | 8.3 Poor communication in relationships |  |
| **9. Future Planning or Information Support** | 9.1 Future Planning ANY |  |
|  | 9.1 Future planning support |  |
|  | 9.2 Other information support |  |
| **10. Changing / dynamic need** | 10.1 Changing / dynamic need ANY |  |
|  | 10.2 End of life care |  |
|  | 10.2 Short prognosis |  |
|  | 10.3 Change in condition |  |
| **11. Family /Carer Support Need** | 11.1 Family / carer Need ANY |  |
|  | 11.2 Emotional, psychological, spiritual need of carer |  |
|  | 11.3 Other need of carer |  |
| **12. Healthcare Provider/ Service** | 12.1 Healthcare provider/ service ANY |  |
|  | 12.2 HCP lack of expertise (self identified) |  |
|  | 12.3 Dissonance between patient and healthcare provider |  |
|  | 12.4 Negative experiences of patient in healthcare services |  |
| **13. Patient/ Family Preferences** | 13.1 Patient / family preferences ANY |  |
|  | 13.2 PPD/ PPC hospice |  |
|  | 13.3 Expressed preference for service involvement |  |
| **14. Other Reason** | (State what) |  |

**Supplementary material 2: Focus group guide**

Welcome

Introductions & any housekeeping points

Introduction

- Role to guide and facilitate discussion within the group, with aid of questions and prompts
- Session is being audio recorded, some notes may also be taken during the session to help remember what has been said.
- Please feel free to share opinions and thoughts, there are no right or wrong answers, and the aim of the discussion is to hear your experiences and thoughts.
- We would ask that you avoid identifying patients, families or staff members during the discussion.
- Please remember you are free to withdraw and leave the session at any point.
- I will begin by presenting some of the data collected in the first stage of this project, and then begin the discussion.
- Any questions/ ready to begin.

**Data presentation**

Run through of key data points on powerpoint (10 mins)

**Discussion Guide**

**What do you think are the main reasons for referrals to specialist palliative care services?**

- Does the data presented reflect this?

- What things does the data presented not capture?

**Do reasons for referral differ between patients with a cancer or a non-cancer diagnosis?**

- If so, how do the two differ?

**Referral forms vary across sites, what impact do you think this has on referrals?**

- Can show referral forms from different sites, and / or slide comparing these

- How do forms vary? What impact might this have?

**In your experience, how well do referrals communicate the key issues for a patient?**

- How do the issues you identify on first assessment of a patient compare to the referral information?

**If time allows:**

**What is your view about the appropriateness of referrals you generally receive?**

Are any referrals inappropriate? If so why?

**Supplementary material 3: Reporting Guidelines**

**Good Reporting of A Mixed Methods Study” (GRAMMS)^1^**

| **Item** | **Location in manuscript** |
| --- | --- |
| 1. Describe the justification for using a mixed methods approach to the research question | Methods – See paragraph on Design. |
| 1. Describe the design in terms of the purpose, priority and sequence of methods | Methods – See paragraph on Design. |
| 1. Describe each method in terms of sampling, data collection and analysis | This information for both the retrospective case note review and focus groups is contained in the methods section |
| 1. Describe where integration has occurred, how it has occurred and who has participated in it | See methods - design |
| 1. Describe any limitation of one method associated with the present of the other method | See Discussion - limitations |
| 1. Describe any insights gained from mixing or integrating methods | See Discussion |

^1^ O’Cathain A, Murphy E, Nicholl J. The quality of mixed methods studies in health services research. J Health Serv Res Policy. 2008 Apr;13(2):92–8
